# Impact of the Copper Second Coordination Sphere on Catalytic Performance and Substrate Specificity of a Bacterial Lytic Polysaccharide Monooxygenase

**DOI:** 10.1021/acsomega.4c02666

**Published:** 2024-05-15

**Authors:** Kelsi R. Hall, Maja Mollatt, Zarah Forsberg, Ole Golten, Lorenz Schwaiger, Roland Ludwig, Iván Ayuso-Fernández, Vincent G. H. Eijsink, Morten Sørlie

**Affiliations:** † Faculty of Chemistry, Biotechnology and Food Science, Norwegian University of Life Sciences (NMBU), Ås 1432, Norway; ‡ School of Biological Sciences, University of Canterbury, Christchurch 8140, New Zealand; § Department of Food Science and Technology, Institute of Food Technology, University of Natural Resources and Life Sciences, Vienna, BOKU 1190 Vienna, Austria

## Abstract

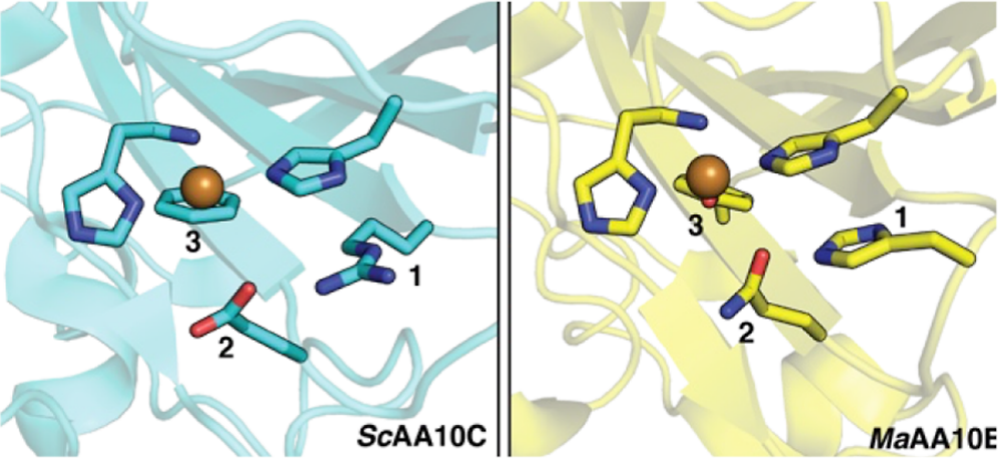

Lytic polysaccharide monooxygenases (LPMOs) catalyze the oxidative cleavage of glycosidic bonds in recalcitrant polysaccharides, such as cellulose and chitin, using a single copper cofactor bound in a conserved histidine brace with a more variable second coordination sphere. Cellulose-active LPMOs in the fungal AA9 family and in a subset of bacterial AA10 enzymes contain a His-Gln-Tyr second sphere motif, whereas other cellulose-active AA10s have an Arg–Glu–Phe motif. To shine a light on the impact of this variation, we generated single, double, and triple mutations changing the His^216^–Gln^219^–Tyr^221^ motif in cellulose- and chitin-oxidizing *Ma*AA10B toward Arg–Glu–Phe. These mutations generally reduced enzyme performance due to rapid inactivation under turnover conditions, showing that catalytic fine-tuning of the histidine brace is complex and that the roles of these second sphere residues are strongly interconnected. Studies of copper reactivity showed remarkable effects, such as an increase in oxidase activity following the Q219E mutation and a strong dependence of this effect on the presence of Tyr at position 221. In reductant-driven reactions, differences in oxidase activity, which lead to different levels of in situ generated H_2_O_2_, correlated with differences in polysaccharide-degrading ability. The single Q219E mutant displayed a marked increase in activity on chitin in both reductant-driven reactions and reactions fueled by exogenously added H_2_O_2_. Thus, it seems that the evolution of substrate specificity in LPMOs involves both the extended substrate-binding surface and the second coordination sphere.

## Introduction

The breakdown of polysaccharides is central to many biological processes and involves multiple enzymes, including lytic polysaccharide monooxygenases (LPMOs). These powerful monocopper enzymes are capable of oxidizing C–H bonds at the C1 and/or C4 carbon of glycosidic linkages in a broad range of polysaccharide substrates, including cellulose,^1−3^ chitin,^4^ various types of hemicelluloses,^5,6^ and starch.^7,8^ Recently, these enzymes have also been shown to play a role in microbial pathogenesis^9,10^ and cellular development.^11−14^

In the CAZy database, LPMOs are categorized into eight of the 17 auxiliary activity families (AA9–11 and AA13–AA17) based on sequence similarities.^15^ Despite large differences between the sequences of LPMOs in these different families, there are several conserved features evident in the secondary structure that unify all LPMOs. The core of these enzymes contains an immunoglobulin-like β-sandwich fold, generally consisting of two β-sheets, which are connected by several loops and helices.^16,17^ Most LPMOs have rather planar substrate-binding surfaces^18^ containing an exposed monocopper active site. The active site comprises a universally conserved histidine brace where the copper is coordinated by three nitrogen ligands.^2,17^ Despite the presence of this conserved histidine brace, it alone is not responsible for LPMO catalysis. Both structural^19^ and mutational^20−22^ studies suggest that second sphere residues, not directly coordinating the copper, have a major impact on LPMO reactivity.

The catalytic mechanism of LPMOs is not yet fully understood; however, in recent years progress has been made with the discovery that H_2_O_2_ is the preferred cosubstrate rather than molecular oxygen.^23−27^ Today, the prevailing view on the reaction catalyzed by LPMOs entails that the LPMO-Cu(II) is first reduced to LPMO-Cu(I) by a priming reduction step, followed by binding of H_2_O_2_ and homolytic cleavage.^21,24,26,28−30^ This is believed to generate a Cu-bound hydroxide species and a hydroxyl radical where the latter abstracts a hydrogen from the Cu-bound hydroxide to generate a Cu(II)-oxyl species, the formation of which is generally accepted.^21,28,31^ The Cu(II)-oxyl species then abstracts a hydrogen from the C–H bond of either C1 or C4 of the carbohydrate substrate, followed by hydroxylation of the bond, ultimately destabilizing the bond and leading to glycosidic bond cleavage.^3,28,32,33^ This mechanism relies on precise confinement of the reactive species to ensure targeted substrate hydroxylation and to prevent autocatalytic damage to the enzyme.^21,26,31^ Substrate-binding is a major contributor to such confinement, shielding the copper site from the solvent, as are residues in the secondary coordination sphere that interact with emerging reactive oxygen species.^21,34,35^

**Figure 1:**
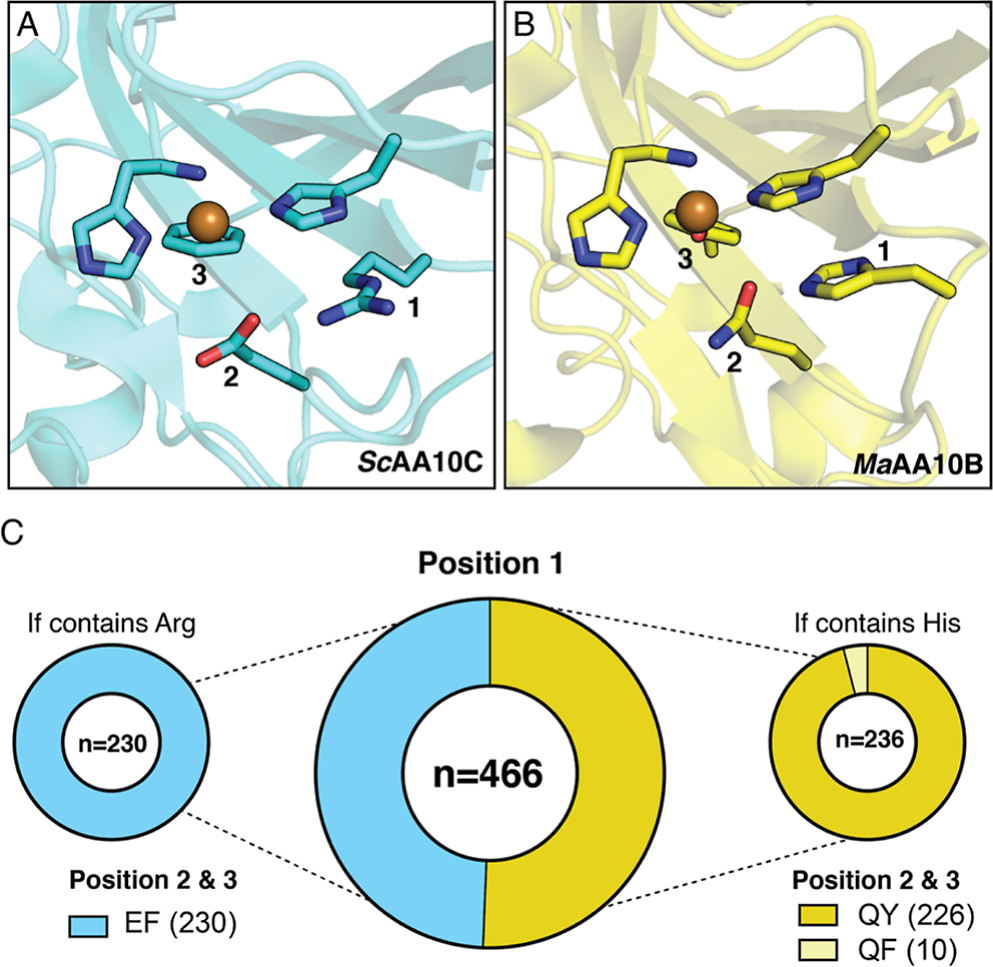
Prevalence of second sphere residues in cellulose-active AA10 LPMOs. Panels A and B show the second sphere arrangements in *Sc*AA10C (PDB: 4OY7) and *Ma*AA10B (PDB: 5OPF), respectively. For clarity, the residues at positions 1, 2, and 3 are numbered. The copper-coordinating histidine brace is also shown. (C) All cellulose-active and mixed activity AA10 LPMOs from the CAZy database (*n* = 466) were aligned to determine the frequency of different amino acids at positions 1, 2, and 3. The amino acid combinations found in *Ma*AA10B and *Sc*AA10C are shown in dark yellow and blue, respectively. Functionally characterized LPMOs with the REF motif show activity on cellulose, whereas characterized LPMOs with the HQY motif show activity on both cellulose and chitin (“mixed” activity). To the best of our knowledge, LPMOs containing the HQF motif (light yellow) have not yet been functionally characterized.

Although similarities exist in the secondary coordination spheres, differences are evident between and within the different LPMO families. All LPMOs contain either a tyrosine or phenylalanine residue in a buried position axial to the copper, with tyrosine prevalent in most LPMO families but not in the AA10 family where this residue typically is a phenylalanine (Figure 1). Another conserved second sphere residue is a glutamine or glutamate residue, which typically coexists with an axial tyrosine or phenylalanine, respectively, in AA9 and AA10 LPMOs. This glutamine/glutamate residue has been shown to play a crucial role in the peroxygenase reaction through constraining and orienting H_2_O_2_ and subsequent reactive intermediates.^21,31,36^ Mutagenesis studies have confirmed this, along with an additional role in controlling copper reactivity.^21,22^ Alongside the glutamine in AA9s, a conserved histidine residue has also been predicted to play a role in positioning oxygen species and has a debated role as a proton donor.^19,37−39^

Bacterial LPMOs in the AA10 family are active on either chitin or cellulose, while a small subset of enzymes in this family can oxidize both substrates. One example of the latter is one of the LPMOs from *Micromonospora aurantiaca*, *Ma*AA10B.^40^ In addition to its dual substrate specificity, this enzyme stands out from the majority of AA10 LPMOs by having a secondary coordination sphere that resembles an AA9, due to the presence of histidine, glutamine, and tyrosine. Other cellulose-active AA10s, such as *Sc*AA10C,^41^ contain arginine, glutamate, and phenylalanine (Figure 1). In this study, we explored the role of these three second sphere residues (His^216^–Gln^219^–Tyr^221^) in *Ma*AA10B. To investigate how the variability of these second sphere residues affects LPMO reactivity and substrate specificity, the equivalent second sphere residues found in *Sc*AA10C (Arg–Glu–Phe) were introduced into *Ma*AA10B, alone or in combination. The results show that these residues play a pivotal role in modulating LPMO reactivity and substrate specificity.

## Experimental Procedures

### LPMO Sequence Space and Analysis of Second Sphere Residues

AA10 LPMO sequences were retrieved from the dbCAN2 database^42^ (07262023 version), which integrates HMMER and DIAMOND searches for automated annotation of carbohydrate-active enzymes in available genomes. The in-house script dbcan_curation.sh was used to fetch unique AA10 LPMOs and remove long and short sequences. After multiple sequence alignment with MAFFT (FFT-NS-1 option, fast but rough alignment),^43^ the signal peptides and carbohydrate-binding modules (CBMs) were removed, leaving the copper-binding N-terminal histidine as the first amino acid of all proteins in the data set that now only contained catalytic domains. The phylogenetic tree was built using fasttree with default parameters after another round of alignment with MAFFT (L-INS-i option, more accurate)^44^ (Figure S1). AA10 LPMOs were functionally annotated and clustered according to the three known types of substrate specificity they have, chitin-active, cellulose-active, or mixed chitin-/cellulose-active, corresponding to well-differentiated clades in the phylogenies (Figure S1). Sequence subsets from clade II corresponding to C1 cellulose-active (230 sequences) and C1 chitin-active/C1–C4 cellulose-active (236 sequences) LPMOs were manually selected from the phylogeny. These subsets were further split into CBM-containing LPMOs (267 sequences) or LPMOs existing as catalytic domains only (199 sequences). Combinations of four key residues in the copper second sphere of coordination were analyzed for each of the subsets after realignment of the sequences with MAFFT (L-INS-i option). In the sequence of *Sc*AA10C (UniProt Q9RJY2), the four key positions are Arg^212^, Asp^214^, Glu^217^, and Phe^219^. The script to analyze combinations of amino acids in the AA10 LPMOs sequence space (count_amino_acid_combinations.py) can be applied to any protein family. These scripts are available at https://github.com/IAyuso.

### Cloning and Site-Directed Mutagenesis

For all single mutants (HQF, HEY, RQY) and one double mutant (HEF), a one-step polymerase chain reaction (PCR) method was used to simultaneously amplify the pRSETB backbone and the *Ma*AA10B gene while also introducing the desired mutations. The codon-optimized gene encoding the *M. aurantiaca* ATCC 27029 LPMO (*Ma*AA10B; residues 1–366; UniProtKB D9SZQ3; signal peptide-AA10-linker-CBM2), cloned in a previous study,^40^ was used as a template. Primers were designed according to Qi and Scholthof (2008)^45^ and included an 18-nucleotide 5′ region complementary to the other primer and a unique 25 nucleotide 3′ region (Table S1). The desired mutations were included in the middle of the 18-nucleotide complementary region. Q5 high-fidelity 2 × master mix was used to perform the PCR as per the manufacturer’s instructions (NEB, Ipswich, MA, USA), and the resulting product was treated with *Dpn*I prior to transformation. For the remaining double (RQF, REY) and triple mutants (REF, REFex), genes encoding the catalytic domains and including 25 bp overhangs complementary to the pRSETB backbone and the CBM (CBM2) were ordered from Thermo Fisher Scientific (Waltham, MA, USA). The pRSETB backbone including the part of the LPMO gene encoding the CBM2 was amplified using gene specific primers (Table S1). Two × NEBuilder HiFi DNA assembly (NEB, Ipswich, MA, USA) was used to join the backbone and gene fragment together. Sequence-verified plasmids were used to transform chemically competent *E. coli* BL21(DE3) cells (Thermo Fisher Scientific, Waltham, MA, USA), and transformants were used for protein expression.

### Expression and Purification

Protein expression and purification were performed as described previously^40^ with slight modifications. Freshly transformed *E. coli* BL21(DE3) cells harboring a LPMO-encoding plasmid were used to inoculate two 500 mL flasks containing Terrific Broth supplemented with 100 μg/mL ampicillin. Cells were grown at 37 °C for 20 h in 1 L bottles attached to an aeration system. Cells were harvested by centrifugation, and the protein was extracted from the periplasm using osmotic shock. The resulting extract was sterilized through a 0.45 μm filter and adjusted to 25 mM bis-tris propane, pH 9.5. The adjusted extract was loaded onto a 5 mL HiTrap DEAE FF column (Cytiva, Marlborough, MA, USA), equilibrated with 25 mM bis-tris propane, pH 9.5. Under these conditions, the *Ma*AA10B variants eluted in the flow through, appearing relatively pure. Fractions containing *Ma*AA10B were assessed using SDS PAGE and were pooled and concentrated using Amicon Ultra-15 Centrifugal filters (Merck, Burlington, MA, USA) with a 10 kDa cut off. The concentrated protein sample was loaded onto a HiLoad 16/60 Superdex 75 size exclusion column (Cytiva, Marlborough, MA, USA) equilibrated with 50 mM Tris-HCl, pH 7.5, 200 mM NaCl. Protein purity was assessed by SDS PAGE and fractions containing the correct-sized protein were pooled. A 2 × molar excess of Cu(II)SO_4_ was added to the pooled protein sample followed by incubation on ice for 60 min. Excess copper and salt were removed, and the protein was simultaneously exchanged into 20 mM sodium phosphate, pH 6.0, using an Amicon Ultra-15 Centrifugal filter (Merck, Burlington, MA, USA) with a 10 kDa cut off. Full-length *Sc*AA10C and CBM2-truncated *Ma*AA11B were expressed and purified as described previously.^40,41^ The final protein concentrations were determined by measuring the A_280_ and using the theoretical extinction coefficients (Table S2), calculated using the ExPASy ProtParam tool (https://web.expasy.org/protparam/).

### Cellulose and β-Chitin Degradation Assays

Standard reactions contained 1 μM LPMO, 20 mM sodium phosphate, pH 6.0, and either phosphoric acid swollen cellulose (PASC) (0.1–0.5% (w/v); prepared from Avicel as described previously^46^) or deproteinized β-chitin (0.1–1% w/v) extracted from squid pen (batch 20140101, France Chitin, Orange, France). Reactions were initiated with the addition of ascorbate (1 mM) and incubated at 40 °C, 1000 rpm in an Eppendorf ThermoMixer C (Eppendorf, Hamburg, Germany) for up to 24 h. At regular intervals, 60 μL of samples were taken, and activity was stopped by separating the enzyme from the insoluble substrate using a 0.45 μm 96-well filter plate (Millipore, Burlington, MA, USA) and a Millipore vacuum manifold. In reactions containing exogenous H_2_O_2_, 100 μM H_2_O_2_ was added to the reactions prior to the addition of ascorbate. The concentration of the H_2_O_2_ stock solution was determined by measuring the absorbance at 240 nm and using an extinction coefficient of 43.6 M^–1^cm^–1^. To allow for quantification of oxidized products, samples with cellulose-derived soluble products were treated with endoglucanase from *Thermobifida fusca* (*Tf*Cel6A, produced in-house^47^) to a final concentration of 1 μM and incubated at 37 °C overnight to convert soluble C1-oxidized products to a mixture of oxidized dimers and trimers (GlcGlc1A and Glc_2_Glc1A). For C4-oxidized products, the levels were very low for most of the protein variants characterized in this study and these products were not quantified, therefore the formation of C1-oxidized products was used to indicate LPMO activity. Samples with chitin-derived soluble products were treated in the same manner with a chitobiase (*Sm*CHB, produced in-house^48^) to convert soluble chitooligomers to *N*-acetylglucosamine (GlcNAc, A1, native) and chitobionic acid (GlcNAcGlcNAc1A, A2^ox^).

### Quantification of Cellulose-Derived Oxidized Products

Quantification of C1-oxidized dimers and trimers (GlcGlc1A and Glc_2_Glc1A) was performed using high-performance anion exchange chromatography with pulsed amperometric detection (HPAEC-PAD), using a 26 min gradient as described previously.^49^ HPAEC-PAD was performed with a Dionex ICS6000 (Thermo Fisher Scientific, Waltham, MA, USA) equipped with a 1 × 250 mm Dionex CarboPac PA-200 analytical column attached to 1 × 50 mm Dionex CarboPac PA-200 guard column. The operational flow was at 63 μL/min, and 4 μL samples were injected. Eluent generator cartridges were used containing methanesulfonic acid (MSA) and potassium hydroxide (KOH) to produce potassium methanesulfonate salts (KMSA). To produce C1-oxidized standards, native cellobiose and cellotriose (Megazyme, Bray, Ireland) were mixed to a final concentration of 0.5 mM and incubated at 40 °C overnight with 2 μM cellobiose dehydrogenase from *Myriococcim thermophilum* (*Mt*CDH, produced in-house^50^). Control reactions without ascorbate were included for all enzyme variants but are omitted from figures for clarity as significant product formation was never observed.

### Quantification of Chitin-Derived Oxidized Products

Quantification of chitin-derived soluble LPMO products was assessed by quantifying the native monomer (A1 native, GlcNAc) formed after treatment with chitobiase (see above). Analysis was performed using a Dionex rapid separation LC (RSLC) system equipped with a 100 × 7.8 mm Rezex RFQ-Fast Acid H+ (8%) (Phenomenex) column at 85 °C. For analysis, 8 μL samples were injected and eluted using a 6 min isocratic gradient of 5 mM sulfuric acid at a flow rate of 1 mL/min. The eluted products were monitored using a 194 nm UV detector. Quantification was performed using *N*-acetyl-glucosamine (A1 native, Megazyme, Bray, Ireland) as the standard. The oxidized dimer (A2^ox^) was also observed but could not be reliably quantified as product levels generally were low and the peak overlapped with a signal corresponding to ascorbate. The initial rates of product formation were corrected for A1 native product detected in control reactions without LPMO. Control reactions without ascorbate were included for all enzyme variants but are omitted from figures for clarity as significant product formation was never observed.

### H_2_O_2_ Production Assay

H_2_O_2_ production was measured as previously described.^51^ Amplex Red Reagent (Thermo Fisher Scientific, Waltham, MA, USA) was dissolved in dimethyl sulfoxide (DMSO) at a stock concentration of 10 mM. Reactions were prepared in a 90 μL volume containing 2 μM LPMO, 100 μM Amplex Red Reagent, 5 U/mL horseradish peroxidase (HRP), and 20 mM sodium phosphate, pH 6.0. After incubation at 30 °C for 5 min, the reaction was initiated with the addition of 10 μL of 10 mM ascorbate (1 mM final concentration). Formation of resorufin was monitored at 540 nm over 40 min in a Multiskan FC microplate photometer (Thermo Fisher Scientific, Waltham, MA, USA). A H_2_O_2_ standard curve was prepared in the same manner, with ascorbate added prior to the addition of HRP and Amplex Red Reagent. The apparent initial rate of H_2_O_2_ production was calculated from the linear portion of the progress curves. Formation of H_2_O_2_ in reactions containing the substrate [0.2% (w/v) PASC or β-chitin] was assessed using the same assay, with the following modifications. The reactions were incubated at 40 °C, 1000 rpm, in an Eppendorf ThermoMixer C (Hamburg, Germany) and at regular intervals 100 μL samples were taken after which the insoluble substrate was removed using a 0.45 μm filter plate. The amount of resorufin in the resulting supernatant was immediately measured by determining absorbance at 540 nm. A H_2_O_2_ standard curve was generated as described above but now also containing 0.2% (w/v) PASC or β-chitin.

### Binding to PASC and β-Chitin

Substrate binding was assessed in reaction mixtures containing 0.5% (w/v) PASC or 1% (w/v) β-chitin and 3 μM LPMO (full-length or truncated WT *Ma*AA10B) in 20 mM sodium phosphate buffer, pH 6.0. The reactions were incubated at 40 °C in an Eppendorf Comfort Thermomixer set to 1000 rpm. At various time points (2.5, 5, 15, and 30 min), a sample was taken and filtered using a 0.45 μm filter plate and a Millipore vacuum manifold to remove the insoluble substrate and substrate-bound protein. The relative amount of protein in the supernatant was determined by measuring the A_280_.

### Real-Time Monitoring of H_2_O_2_ Consumption

The consumption of H_2_O_2_ in the presence of PASC or β-chitin was measured using an electrochemical sensor for real-time monitoring of H_2_O_2_ levels, as described by Schwaiger et al., (2024).^52^ The method utilizes a Prussian blue-modified gold rotating disc electrode, which was prepared as previously described.^52^ Reactions contained 20 mM sodium phosphate, pH 6.0, 100 mM KCl, 1 μM LPMO, 0.1% (w/v) PASC or 1% (w/v) β-chitin, and 100 μM H_2_O_2_ in a 4 mL reaction volume. The H_2_O_2_ was added in five sequential steps while monitoring the signal, thus creating an internal standard curve for the H_2_O_2_ concentration. After adding H_2_O_2_ in five steps, the LPMO reaction was initiated by adding ascorbate to a final concentration of 1 mM. The electrode was rotated at an angular velocity of 50 s^–1^ in an electrochemical reaction chamber kept at 40 °C.

## Results and Discussion

### Frequency of Second Sphere Residue Arrangements in Cellulose-Active AA10 LPMOs

Cellulose-active AA10 LPMOs show different second sphere architectures.^40^ For example, the active sites of *Ma*AA10B and *Sc*AA10C contain 3 s sphere residues whose side chains differ but have spatially conserved locations: a histidine or arginine (position 1), a glutamine or glutamate (position 2), and a tyrosine or phenylalanine (position 3) (Figure 1). Apart from tyrosine/phenylalanine, these residues are situated on the protein surface. We set out to investigate how prevalent these second sphere residues are in all predicted cellulose-active AA10 LPMOs present in the CAZy database (*n* = 466; this set includes LPMOs with “mixed”) (i.e., cellulose and chitin, activity; Figure S1). The analysis revealed that two patterns or “motifs” (REF and HQY/HQF) exist in 100% of the cellulose- and mixed-active LPMOs. One motif consisting of arginine, glutamate, and phenylalanine, and hereafter referred to as REF, is evident in 49% of the LPMOs, including *Sc*AA10C. All cellulose-active AA10 LPMOs that contain an arginine at position 1 contain a glutamate and phenylalanine at positions 2 and 3, respectively. The other motif consisting of histidine, glutamine, and tyrosine or phenylalanine, hereafter referred to as HQY and HQF, respectively, is evident in the remaining 51% of the predicted cellulose-active LPMOs, including *Ma*AA10B. In this second group, the HQY motif is strongly dominating (96% HQY, 4% HQF) and is analogous to that commonly found in cellulose-active fungal AA9 LPMOs.

An additional analysis was performed to see if the presence or absence of a CBM correlated with a particular motif (Figure S2). Among LPMOs containing an HQY motif, 69% consisted of a catalytic domain only, while 85% of the LPMOs with a REF motif had a CBM. While these observations show a clear trend linking the REF motif to the presence of a CBM, alternative arrangements are common, i.e., HQY combined with a CBM or REF occurring in LPMOs with a catalytic domain only.

### Site-Directed Mutagenesis of *Ma*AA10B

To address the impact of these second sphere residues, the HQY motif in *Ma*AA10B was mutated, replacing residues with the corresponding residues found in *Sc*AA10C that carries the REF motif. All possible single (*n* = 3) and double (*n* = 3) mutants were assessed as well as two variants of a triple mutant (*n* = 2; more details below). A summary of all variants used in this study is shown in Table 1. For simplicity, all *Ma*AA10B variants were assigned a three-letter code signifying the residues present at positions 1, 2, and 3. When a position has been mutated, the relevant position in the three-letter code is underlined. For example, HEY indicates the *Ma*AA10B variant containing the Q219E mutation at position 2.

**Table 1:** Overview of the *Ma*AA10B Variants (+W**T***Sc*AA10C) Used in This Study^a^

	variant code	position 1	position 2	position 3	mutations introduced
	WT *Ma*AA10B HQY	His (H)	Gln (Q)	Tyr (Y)	N/A
single mutants	HQF	His (H)	Gln (Q)	Phe (F)	Y221F
	HEY	His (H)	Glu (E)	Tyr (Y)	Q219E
	RQY	Arg (R)	Gln (Q)	Tyr (Y)	A214R/H216G^b^
double mutants	HEF	His (H)	Glu (E)	Phe (F)	Q219E/Y221F
	RQF	Arg (R)	Gln (Q)	Phe (F)	A214R/H216G^b^/Y221F
	REY	Arg (R)	Glu (E)	Tyr (Y)	A214R/H216G^b^/Q219E
triple mutants	REF	Arg (R)	Glu (E)	Phe (F)	A214R/H216G^b^/Q219E/Y221F
	REFex	Arg (R)	Glu (E)	Phe (F)	A214R/H216D/L217S/D218Q/Q219E/Y221F
	WT *Sc*AA10C REF	Arg (R)	Glu (E)	Phe (F)	N/A

a The amino acids present at positions 1, 2, and 3 are listed and were used to generate a unique code for each MaAA10B variant. The positions that were mutated are underlined in the motif code, and the actual mutations are shown in the last column. See text and Supporting Information, Discussion for more details.

b Additional mutation to create space, allowing the desired arginine residue to be introduced (see Figure S3 and Supporting Information, Discussion).

The glutamine to glutamate (position 2; Figure 1) and tyrosine to phenylalanine (position 3; Figure 1) replacements were straightforward as these residues align with one another in the sequences and structures of AA10 LPMOs (Figure S3A). For the histidine to arginine exchange, the situation was more complicated, as while the headgroups of these residues align structurally (Figure S3B), the location of this residue in the main chain varies, as do some adjacent residues that pose steric limitations. Therefore, as detailed in Figure S3 and the associated Supporting Information, Discussion, the histidine to arginine exchange required two mutations in *Ma*AA10B, A214R, and H216G. An additional triple mutant variant, referred to as REFex (denoting REF exchange), was generated in which the complete loop region running from Ala^214^ to Gln^219^, containing positions 1 and 2, was replaced with the corresponding loop region of *Sc*AA10C. Functional characterization of the two triple mutants showed minimal differences with both variants relatively inactive and unstable (see below); hence, this additional design was not implemented in single and double mutants containing the arginine residue.

All *Ma*AA10B-encoding variant genes were cloned into the pRSETB expression vector, expressed, and purified. Expression levels were low for most mutants, but all variants could be purified with yields on the order of 0.2–1 mg of pure protein per liter of culture.

### Activity of *Ma*AA10B Variants under Apparent Monooxygenase Conditions

**Figure 2:**
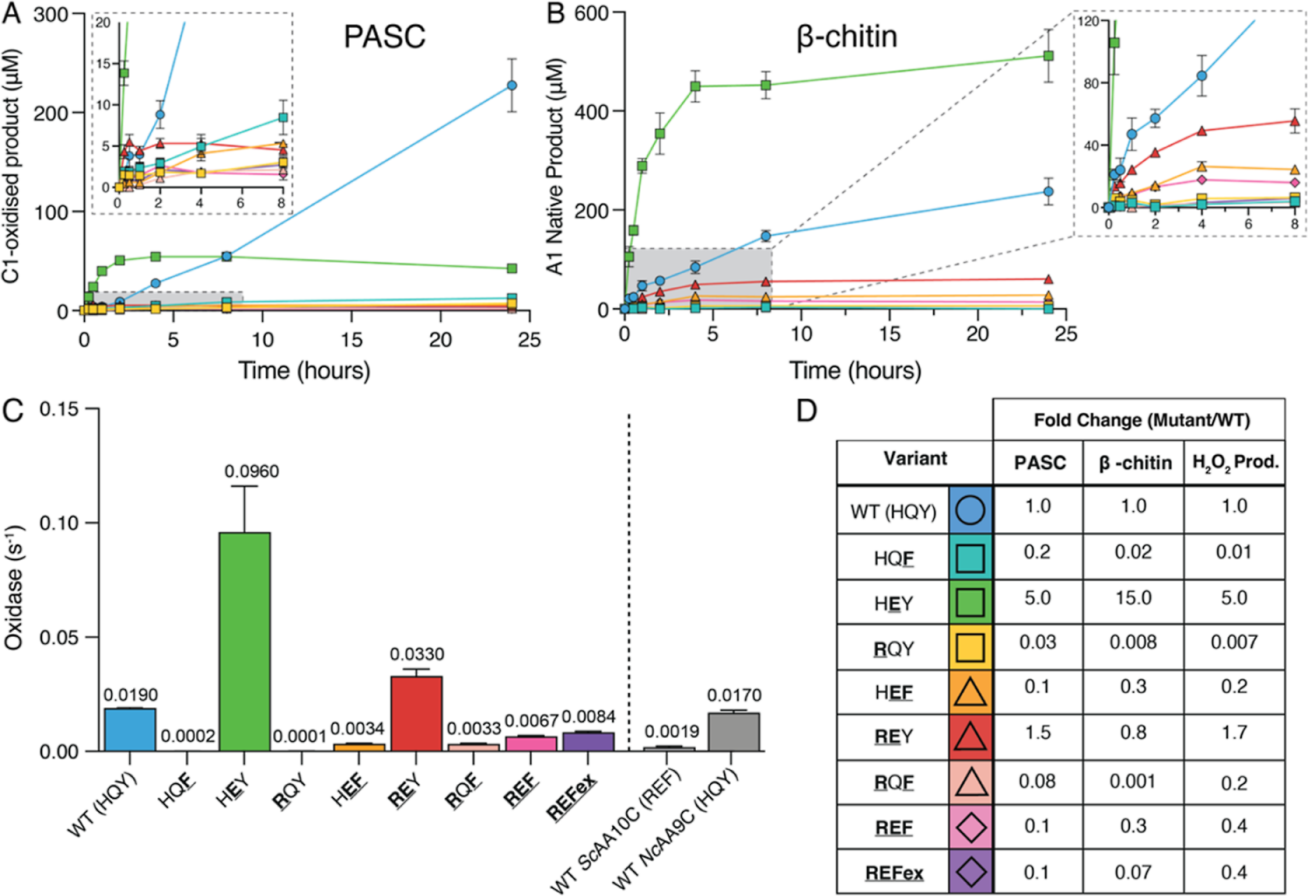
Activity of*Ma*AA10B variants under various conditions. (A) C1-oxidized products released from 0.1% (w/v) PASC. (B) Soluble oxidized products released from 1% (w/v) β-chitin quantified as A1 (see Experimental Procedures section for details). Reactions contained 1 μM LPMO, 1 mM ascorbate, and 20 mM sodium phosphate, pH 6.0, and were performed at 40 °C, 1000 rpm. The zoomed insets show the first 8 h of the reaction. (C) Apparent rate of H_2_O_2_ production (oxidase activity) measured using the Amplex Red/HRP assay. Reactions contained 2 μM LPMO, 1 mM ascorbate, 100 μM Amplex Red, and 5 U/mL HRP in 20 mM sodium phosphate, pH 6.0, and were performed at 30 °C. The concentration of H_2_O_2_ was calculated using a standard curve, which included 1 mM ascorbate to account for side-reactions between ascorbate and Amplex Red. The reported rate is adjusted to 1 μM LPMO. The rate for WT *Nc*AA9C, at pH 6.5, is derived from Rieder et al., (2021).^54^ (D) Fold change in the initial rates of the degradation of PASC or β-chitin and H_2_O_2_ production (oxidase activity) for *Ma*AA10B variants compared to WT *Ma*AA10B. Note that some of these numbers are based on low activities and product levels and may be affected by enzyme inactivation; thus, these fold changes should be considered rough estimates. The values for the initial rates are shown in Table S3. WT *Ma*AA10B (HQY) is shown as a blue circle (○). Single mutants appear as squares (□), double mutants as triangles (Δ), and triple mutants as diamonds (◇). Error bars show the standard deviation of triplicate reactions.

The activity of all variants was assessed using PASC and β-chitin as substrates, under so-called “monooxygenase conditions”. Under these conditions, the reaction is fueled by a reductant,^53^ in this case 1 mM ascorbic acid, and the reaction is limited by the rate of in situ generation of H_2_O_2_, the kinetically relevant cosubstrate of the LPMO. H_2_O_2_ levels in the reaction are determined by the oxidase activity of the LPMO, i.e., turnover of O_2_ by the enzyme to generate H_2_O_2_,^51^ and by abiotic reactions of the reductant with O_2_. If H_2_O_2_ levels become too high^26^ or if the catalytic center in the LPMO-substrate complex does not provide sufficient confinement and control of the emerging reactive oxygen species,^21^ the LPMO may become oxidatively damaged and inactivated. Figure 2A,B shows the degradation of cellulose and chitin by the *Ma*AA10B variants, while Figure 2C shows the oxidase activity (i.e., H_2_O_2_-producing capacity) of these variants in the absence of substrate. Figure 2D shows an overview of the observed mutational effects. Of note, *Ma*AA10B variants-catalyzed oxidation of chitin is quantified by determining the *N*-acetyl-glucosamine (A1) concentration, the product of treatment by a chitobiase, which is only capable of hydrolyzing soluble, oxidized oligomers not insoluble chitin, resulting from LPMO action (see Experimental Procedures section for details).

With the exception of the HEY mutant, all *Ma*AA10B variants produced substantially less product than WT *Ma*AA10B on both PASC and β-chitin (Figure 2A,B). The shapes of the product curves indicate substrate-dependent differences between the enzyme variants in terms of the initial rate of the reaction and/or the onset of enzyme inactivation (that causes product formation to level off). Due to these two interconnected effects and the low activity of some of the variants, a full deconvolution of all mutational effects is not possible. The general picture emerging from these results is that most variants have disturbed catalytic centers that no longer allow efficient and stable catalysis. Despite this, some clear and important observations stand out.

The HEY variant, carrying the single Q219E mutation, exhibited the most interesting phenotype, with a faster initial rate of product release for both PASC and β-chitin compared to WT *Ma*AA10B. With PASC, the HEY mutant inactivated much faster than WT *Ma*AA10B, leading to reduced product yields after longer incubation times. This pattern is typical for LPMO reactions containing high amounts of H_2_O_2_. Accordingly, the oxidase activity of the HEY mutant was some five times higher compared to that of WT *Ma*AA10B (Figure 2C). Similar results were obtained previously when mutating the analogous glutamine residue in a cellulose-active AA9 LPMO called *Nc*AA9C.^22^ This fungal LPMO naturally contains the same second sphere motif as *Ma*AA10B, HQY, and the two enzymes have similar oxidase activities (Figure 2C). It is interesting to note that the previously observed impact of this residue on copper reactivity also applies to bacterial AA10 LPMOs, considering the fact that the members of these enzyme families overall share little sequence identity.

Most interestingly, with β-chitin, the HEY variant clearly outperformed WT *Ma*AA10B, showing a much higher initial rate, little enzyme inactivation, and higher total product yields (Figure 2B). Thus, while differences in substrate specificity have previously been attributed to variations in the extended flat substrate-binding surfaces of LPMOs,^55^ the phenotype of the Q219E mutant of *Ma*AA10B leads to the important conclusion that second sphere residues also affect substrate preference. In this respect, it is worth noting that LPMOs in the AA10 and AA11 families that are thought to exclusively degrade chitin have a glutamate in this equivalent position.

Figure 2C shows that all *Ma*AA10B variants exhibited changes in oxidase activity compared to WT *Ma*AA10B. Overall, the fold-changes in the oxidase activity and the fold-changes in the estimated initial rates of PASC and β-chitin degradation showed similar trends (Figure 2D), albeit with some substrate-dependent variation, as discussed above for the HEY mutant that displays drastically increased oxidase activity.

Single mutants other than HEY showed drastic reductions in oxidase activity and close to negligible rates of PASC and β-chitin degradation. Thus, the individual histidine to arginine and tyrosine to phenylalanine mutations damage the catalytic ability of the LPMO, which is perhaps not surprising, considering the observed coevolution of these residues in the HQY/REF motifs. In this respect, it is worth noting that the oxidase activity of *Sc*AA10C, having an REF motif, was only 10-fold lower compared to *Ma*AA10B, having HQY, whereas the two single mutants show an approximately 100-fold reduction. Furthermore, the low oxidase activity of *Sc*AA10C (with REF) shows that having a glutamate instead of a glutamine at position 2 is not enough to obtain a high oxidase activity.

The properties of the double mutants shed more light on how the interplay between second sphere residues affects LPMO activity and substrate preference. The REY mutant exhibited a 1.7-fold higher oxidase activity than WT *Ma*AA10B, representing a 240-fold increase relative to the RQY single mutant and a 3-fold decrease relative to the HEY single mutant (Figure 2D). This clearly shows that the two residues have interconnected effects on the reactivity of the copper site: the combination of arginine and glutamate in REY increases the oxidase activity from the single mutant (RQY) while the arginine mutation limits the effect of the glutamate mutation in HEY. The REY mutant, with its slightly increased oxidase activity, exhibited faster initial activity on PASC (Figure 2A,D; Table S3), whereas, surprisingly, the initial activity on β-chitin was slightly reduced, despite the presence of the glutamate at position 219. This shows that position 1 in the second sphere (arginine or histidine) also affects substrate specificity. The phenotype of REY, relative to HEY, suggests that an arginine is unfavorable for activity on β-chitin and aligns with the fact that such an arginine is lacking from most, but not all,^10,56,57^ chitin-active AA10 LPMOs, likely because it causes steric hindrance for binding of chitin, as discussed by Vaaje-Kolstad et al., (2012).^58^ Still, it seems unlikely that the differences observed between histidine and arginine at position 1 only result from steric effects, since the headgroups of these two residues are predicted to occupy approximately the same position in the protein (Figure S3) and since the REF motif does occur naturally in some chitin-active AA10 LPMOs.

The HEF double mutant exhibited a lower oxidase activity and lower activity on PASC and β-chitin relative to WT *Ma*AA10B. Importantly, this shows that the drastic effect of the Q219E mutation at position 2, as in HEY, is strongly dependent on the presence of a tyrosine at position 3, revealing another example of the interplay between the three targeted second sphere residues. These positions are within hydrogen bonding distance with 2.6 Å between the glutamine headgroup and the hydroxyl group of the tyrosine. This observation is in accord with the similarly low oxidase activity of *Sc*AA10C, which has a phenylalanine at position 3. As is to be expected based on the phenotypes above, e.g., for the single mutants HQF and RQY, the final double mutant, RQF, showed low oxidase activity and low activity on PASC and β-chitin, relative to WT *Ma*AA10B.

The triple mutants, REF and REFex also showed reduced oxidase and polysaccharide oxidizing abilities compared to WT *Ma*AA10B. The triple mutants were more active than some of the single or double mutants, especially for β-chitin, which likely relates to the beneficial effect of the glutamate at position 2 and the clear interplay between the three mutated residues. The fact that the REF mutant, containing the second sphere motif from *Sc*AA10C (an exclusively cellulose-active LPMO), is still able to degrade β-chitin shows that these residues alone do not determine substrate specificity. This aligns well with the above-mentioned study by Jensen et al., (2019)^55^ who showed that *Sc*AA10C could be made chitin-active by mutating residues on the substrate-binding surface beyond the second sphere.

All in all, the data presented in Figure 2 shows that the targeted residues have a major impact on copper reactivity and LPMO activity under apparent monooxygenase conditions and that the effects of the individual residues depend on each other. The impact of the Q219E mutation at position 2 is remarkably context-dependent, particularly on the presence of a tyrosine or a phenylalanine at position 3. Furthermore, the results show that these second sphere residues, and in particular the glutamine/glutamate at position 2, are codeterminants of substrate specificity.

### Closer Look at the Impact of Substrate

LPMO reactions are commonly performed under apparent monooxygenase reactions, meaning that reactions are reductant-driven and reaction rates depend on the rate of in situ generation of H_2_O_2_. Quantitative interpretation of such reactions has many pitfalls. It is well-known that substrate-binding protects LPMOs against oxidative damage^26,59^ by promoting productive rather than off-pathway reactions. Furthermore, substrate-binding decreases LPMO-catalyzed production of H_2_O_2_,^51,60−62^ which may not only affect LPMO activity but also stability (since high H_2_O_2_ levels are potentially damaging). Thus, the dependency of an LPMO reaction on the concentration of productive substrate-binding sites is complex, and this complexity is increased further by the number of productive substrate-binding sites per gram of cellulose or chitin being unknown. Thus, in the present study, we needed to consider whether the choice of the PASC and β-chitin concentrations used above could explain the remarkable apparent difference in substrate specificity between WT *Ma*AA10B and the HEY mutant.

**Figure 3:**
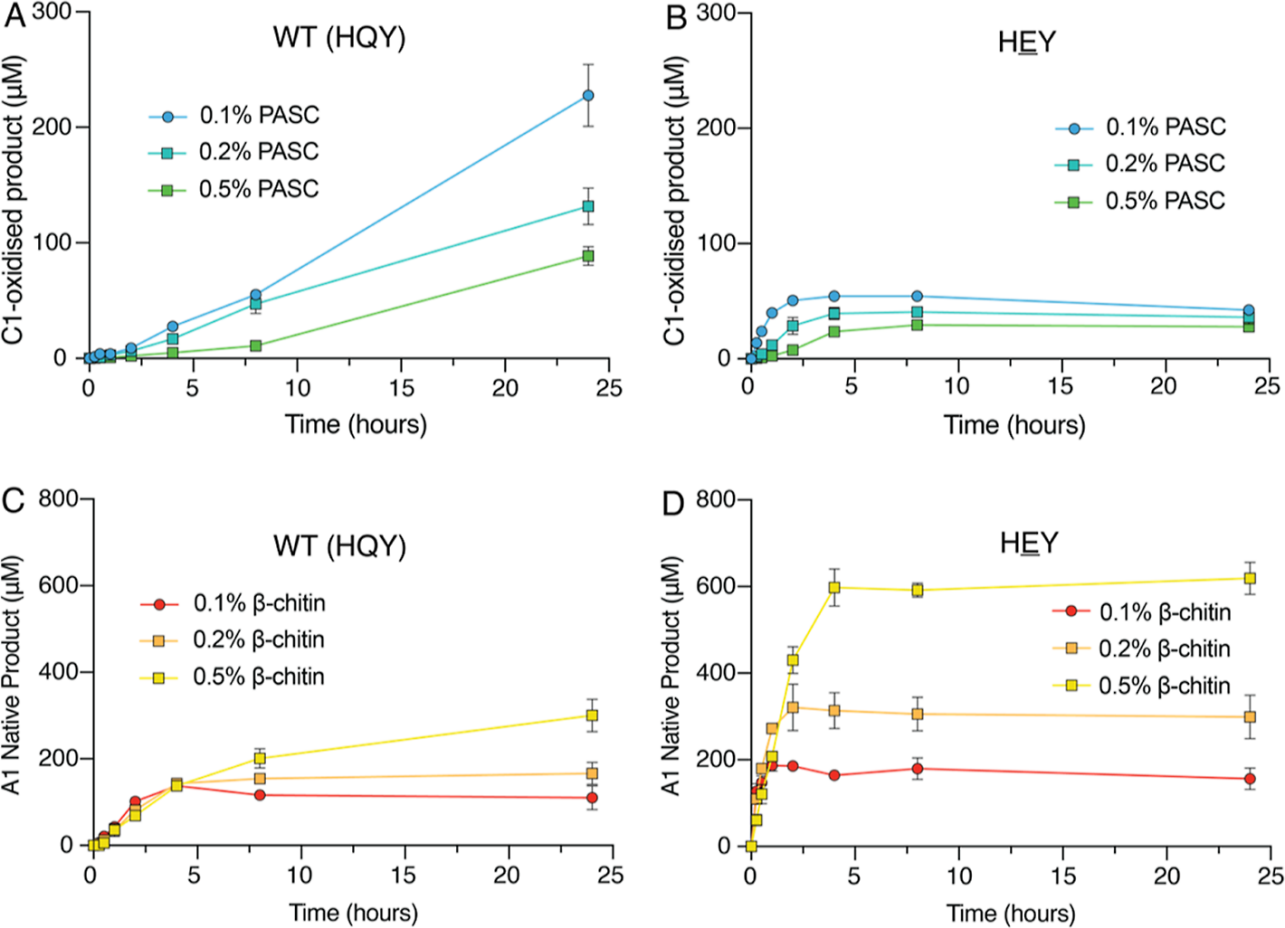
Quantification of soluble oxidized products released under apparent monooxygenase conditions with varying PASC or β-chitin concentrations. Reactions contained 0.1–0.5% (w/v) PASC (A and B) or 0.1–0.5% (w/v) β-chitin (C and D) and 1 μM WT *Ma*AA10B (A and C) or 1 μM HEY (B and D). All reactions were performed at 40 °C, 1000 rpm in 20 mM sodium phosphate, pH 6.0, and initiated with the addition of 1 mM ascorbate. Product formation was monitored after further enzymatic treatment of reaction samples, as described in the Experimental Procedures Section. Error bars show the standard deviation of triplicate reactions.

To investigate the possible effect of the substrate concentration, reactions were performed with 0.1, 0.2, or 0.5% (w/v) PASC or β-chitin under apparent monooxygenase conditions (Figure 3). For all concentrations of PASC and β-chitin, the HEY mutant exhibited a faster initial rate of reaction than WT *Ma*AA10B (Figure 3A,B). Furthermore, for all β-chitin concentrations, the HEY mutant performed better than WT *Ma*AA10B (Figure 3C,D), confirming that the increased activity on β-chitin is due to the Q219E mutation. Thus, the apparent differences in substrate specificity between the two enzyme variants are not, or at least not solely, due to differences in the effective substrate concentration.

**Figure 4:**
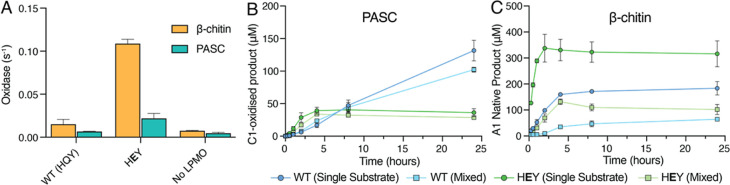
Activity assays to determine the impact of substrate binding. (A) H_2_O_2_ production in the presence of 0.2% (w/v) β-chitin (orange) or 0.2% (w/v) PASC (teal), and substrates alone (no LPMO) as a comparison measured with Amplex Red/HRP. The initial rates of H_2_O_2_ production (s^–1^) were determined from the linear portion of the reaction and corrected for background H_2_O_2_ production in reactions without LPMO. The rates observed in the absence of substrate (see Figure 2C) are 0.019 and 0.096 s^–1^ for WT *Ma*AA10B and the HEY mutant, respectively. Error bars show the standard deviation for duplicate reactions. This measurement is possible because HRP competes efficiently with the LPMO for H_2_O_2_.^27^ Panels B (PASC-derived products) and C (chitin-derived products) show product formation under apparent monooxygenase conditions in reactions containing 0.2% (w/v) PASC (B) or β-chitin (C) or both (B & C). Reactions contained 1 μM LPMO in 20 mM sodium phosphate, pH 6.0, and were incubated at 40 °C, 1000 rpm after initiation with the addition of 1 mM ascorbate. Error bars show the standard deviation of triplicate reactions.

The control experiment depicted in Figure 3 reveals some remarkable differences between the two substrates. For PASC, the activity of WT *Ma*AA10B and the HEY mutant decreased at higher substrate concentrations (Figure 3A,B), which is compatible with an expected inhibitory effect of the substrate on the (reaction rate-limiting) in situ production of H_2_O_2_. Indeed, determination of H_2_O_2_ production in reactions with the substrate showed an approximately 10-fold decrease for WT *Ma*AA10B and a 6-fold decrease for the HEY mutant upon addition of 0.2% (w/v) PASC (Figure 4A; more discussion below). At higher substrate concentrations, rapid inactivation was still evident for the HEY mutant, meaning that the expected increased protection of the enzyme by the substrate did not occur. This suggests that, when acting on cellulose, the HEY mutant has an impaired ability to use H_2_O_2_ productively even when bound to cellulose, as was confirmed by carrying out peroxygenase reactions that are described below.

In striking contrast, for both WT *Ma*AA10B and the HEY mutant, the initial rate of the reaction with β-chitin was hardly affected by increasing the substrate concentration (Figure 3C,D). This could indicate that H_2_O_2_ production by the *Ma*AA10B variants is not as affected by binding to β-chitin. The latter was indeed confirmed by an experiment (Figure 4A) showing that the presence of 0.2% (w/v) β-chitin has only a modest effect on H_2_O_2_ production by WT *Ma*AA10B and essentially no effect on the much higher H_2_O_2_ production by the HEY mutant. The minimal impact of β-chitin, which is truly remarkable in light of studies with cellulose, could indicate that this substrate binds less strongly to the LPMO compared to PASC or could indicate different binding modes, with different impacts on the oxidase activity. Intriguingly, nevertheless, the two enzyme variants are active on chitin.

Binding studies with WT *Ma*AA10B and a truncated variant lacking the CBM2, which is present in all enzyme variants studied here, showed that the CBM is a major determinant of substrate binding (Figure S4). For this reason, and the challenges in producing *Ma*AA10B variants without CBMs, we opted only to perform binding studies with the wild type and its truncated version. Further, the binding data showed relatively fast binding to PASC, whereas binding to β-chitin was weaker and slower. This is in accord with the differences between PASC and β-chitin discussed above. Importantly, the difference was largest for the catalytic domain only, which showed only weak binding to β-chitin. A possible impact of β-chitin on the production of H_2_O_2_ by the LPMO would require the catalytic domain to bind strongly to this substrate, since this would block the copper site and abolish the oxidase reaction. Thus, weak binding of the catalytic domain could explain why, compared to PASC, β-chitin hardly affects H_2_O_2_ production by WT *Ma*AA10B and the HEY mutant. Since the HEY mutant works well on chitin (Figures 2 and 4), a remarkable, counterintuitive, but not unprecedented,^63^ implication of these observations is that the binding affinity of the catalytic domain for various substrates and LPMO activity on these substrates are not necessarily correlated.

To substantiate the remarkable differences between PASC and β-chitin discussed above, we carried out reactions containing a mixture of β-chitin and PASC. The degradation of PASC was hardly affected by the presence of β-chitin for both WT *Ma*AA10B and the HEY mutant (Figure 4B). In contrast, the degradation of β-chitin was clearly inhibited by the presence of PASC (Figure 4C). This result is compatible with the notion that both LPMO variants bind stronger to PASC, which will slow down β-chitin degradation due to substrate competition and because binding of PASC results in decreased H_2_O_2_ production and therefore lower LPMO activity.

### Activity of *Ma*AA10B Variants on PASC and β-Chitin in Reactions Driven by Exogenous H_2_O_2_

In the LPMO reactions described so far, we used standard conditions that are widely used in the field and that lead to reactions being limited by in situ produced H_2_O_2_. To further characterize the mutants, reactions with exogenous H_2_O_2_ were performed. Under these conditions, H_2_O_2_ production by the enzyme, and the variation therein upon mutagenesis, become negligible, allowing assessment of the peroxygenase abilities of each variant. PASC and β-chitin degradation assays were performed with 100 μM H_2_O_2_ and an excess of ascorbate (1 mM) to ensure reduction of the LPMO. Of note, these conditions lead to faster reactions, compared to reductant-driven reactions, while enzyme inactivation will occur since the initial H_2_O_2_ concentration is high.

**Figure 5:**
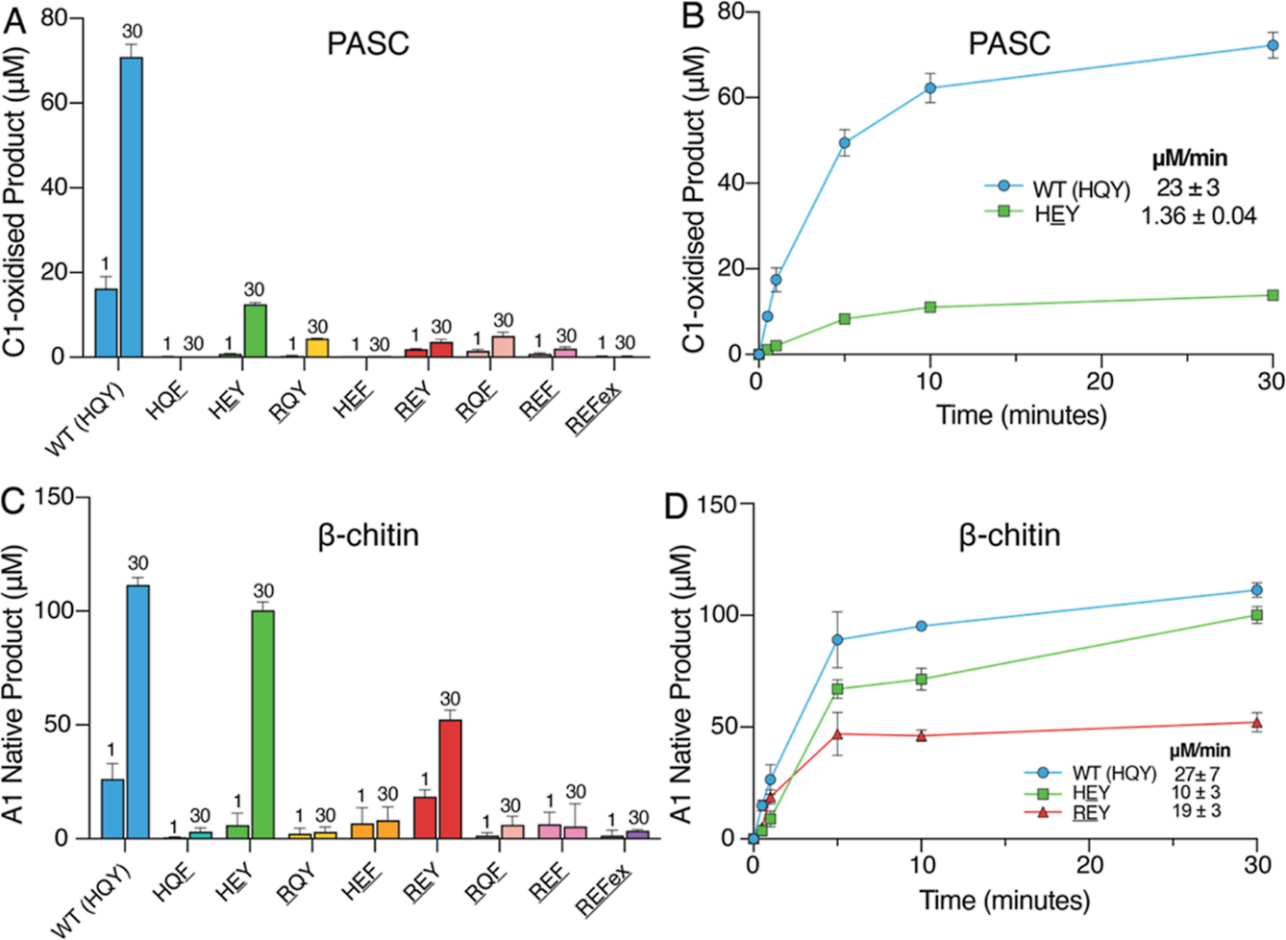
Release of oxidized products by*Ma*AA10B variants in reactions supplemented with H_2_O_2_. (A,B) Oxidized products released from 0.1% (w/v) PASC. (C,D) Oxidized products released from 1% (w/v) β-chitin. Panels A and C show product levels after 1 or 30 min for all variants, whereas panels B and D show time courses for the most active variants (B; WT and HEY. D; WT, HEY, and REY). Approximate initial rates are indicated in panels B and D; note that the rates in these two panels cannot be directly compared because panel B shows the true levels of soluble oxidized sites, while panel D shows monomer levels obtained after enzymatically treating solubilized oxidized oligomers. Reactions contained 1 μM LPMO, 100 μM H_2_O_2_, 1 mM ascorbate, and 0.1% (w/v) PASC or 1% (w/v) β-chitin in 20 mM sodium phosphate, pH 6.0. Reactions were performed at 40 °C, 1000 rpm. Note that the time scale of these reaction is in minutes rather than hours, as in Figures 2, 3, and 4. Error bars show standard deviations of triplicate reactions.

Under these conditions, five of the *Ma*AA10B mutants (HEY, RQY, REY, RQF, REF) produced detectable amounts of oxidized product after reacting on PASC for 30 min, albeit much less than WT *Ma*AA10B (Figure 5A). A full time-course reaction comparing HEY, the best performing mutant in this assay, and WT *Ma*AA10B indicated that lower product formation by the HEY mutant is due to a decrease in enzyme stability and a 17-fold decrease in the apparent initial rate of peroxygenase reaction (Figure 5B). Control experiments in which fresh enzyme was added after 30 min confirmed that the reaction with the HEY mutant slows down due to enzyme inactivation, while the reaction with WT *Ma*AA10B slows down because the externally added H_2_O_2_ has been consumed (Figure S5A,B). All in all, these data show that any mutation or combination of mutations in the HQY motif reduces the peroxygenase activity of *Ma*AA10B on cellulose. The least deteriorating of these mutations, Q219E, yields an increased ability to produce H_2_O_2_ (see above) but reduces the ability to use this H_2_O_2_ productively in reactions with cellulose. Under peroxygenase conditions, this leads to increased enzyme inactivation, reduced apparent initial catalytic rates, and lower product yields (Figure 5B).

With β-chitin, all variants yielded a detectable amount of product, with WT *Ma*AA10B and two of the mutants (HEY and REY) producing a substantial amount (Figure 5C). Rapid enzyme inactivation is apparent for several mutants since product levels after 1 and 30 min are similar. A full time-course reaction with β-chitin comparing WT *Ma*AA10B and the HEY and REY mutants revealed that the arginine-containing variant (REY) was less stable than WT *Ma*AA10B (Figure 5D), supporting the notion that an arginine residue may hamper productive binding of chitin. The HEY mutant performed almost as good as WT *Ma*AA10B (Figure 5D). When acting on β-chitin, this mutant has close to WT-like abilities to use H_2_O_2_ productively. Control experiments showed that both WT *Ma*AA10B and the HEY mutant were inactive at the end of the 30 min incubation period (Figure S5C,D).

**Figure 6:**
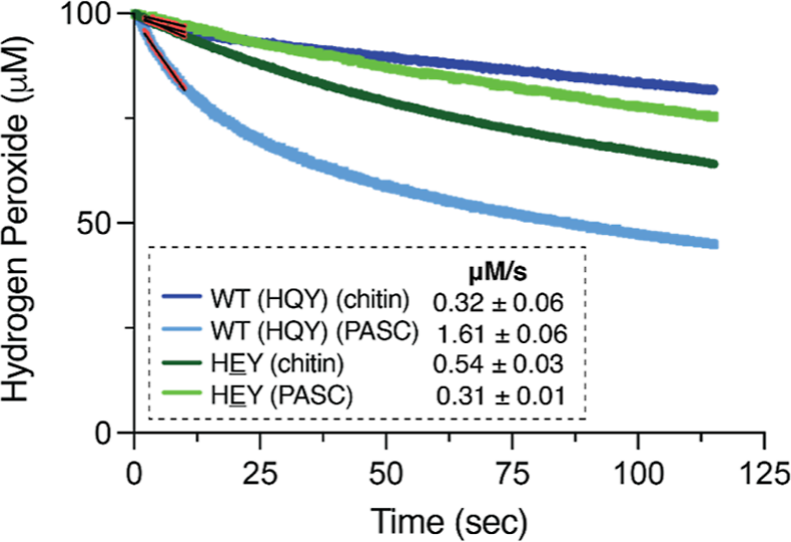
H_2_O_2_ consumption by WT *Ma*AA10B and the HEY mutant in the presence of PASC or β-chitin. H_2_O_2_ consumption was measured in real-time using a Prussian blue-modified rotating gold disc electrode. Reactions contained 1 μM LPMO, 100 μM H_2_O_2_, 1 mM ascorbate, 20 mM sodium phosphate, pH 6.0, 100 mM KCl, and 0.1% (w/v) PASC or 1% (w/v) β-chitin. The electrode was rotated at an angular velocity of 50 s^–1^ in an electrochemical reaction chamber kept at 40 °C. The initial rates of the reactions were estimated from the initial parts of the curves (colored red) and are indicated in the figure. These rates are the average rates derived from three independent reactions with the standard deviation reported.

Quantitative interpretation of the time course reactions shown in Figure 5 is complicated because of the combination of a fast reaction and the occurrence of enzyme inactivation. Also, only soluble products are monitored, which could lead to errors if the mutations have affected the ratio of soluble and insoluble oxidized products. To obtain additional, and more reliable, insight into the possible differences in substrate specificity between the WT *Ma*AA10B and the HEY mutant, we used a recently developed H_2_O_2_ sensor^52^ for real-time measuring of H_2_O_2_ consumption in reactions with PASC and β-chitin (Figure 6). LPMO activity was, hence, assessed by measuring the consumption of the cosubstrate, which is consumed concomitant with the formation of oxidized products in equimolar amounts. This allows the determination of true enzyme kinetics. We opted to determine the initial rates of peroxygenase activity by the wild type and the HEY mutant on both PASC and chitin. First, the wild type displayed higher activity on PASC (1.61 μM/s) than on chitin (0.32 μM/s). Interestingly, the HEY mutant displayed higher activity on chitin (0.54 μM/s) than on PASC (0.31 μM/s), representing a 1.7-fold increase in the initial rate of activity on β-chitin and a 5.2-fold decrease in the initial rate of activity on PASC. These results clearly show the drastic impact of the single Q219E mutation on the substrate specificity of *Ma*AA10B.

## Concluding Remarks

The present mutational study shows that residues in the second coordination sphere of the copper site have a major impact on LPMO functionality, having clear effects on the oxidase activity and on the ability to use H_2_O_2_ productively rather than reacting with H_2_O_2_ in a manner that leads to enzyme damage. Clearly, the roles of the three residues targeted in this study are not independent, which is not surprising considering their coevolution and their proximity to each other, the copper, and the bound substrate. The interplay between the glutamine/glutamate at position 2 and the tyrosine/phenylalanine at position 3 is of particular interest because this link is strong and could be assessed by rather straightforward mutations with likely minimal impact on the protein structure. On this note, the histidine/arginine mutation at position 1 was less straightforward and has a higher risk of generating structural perturbations that may compromise the interpretation of functional consequences. The increased oxidase activity that results from the Q219E mutation at position 2 relies on the presence of a tyrosine at position 3, and future studies should aim to uncover the relationship between these two residues. Interestingly, the interplay between residues at positions 2 and 3 could couple the variation at position 2 to possible protective hole hopping mechanisms mediated by a tyrosine at position 3,^24,64,65^ as recently shown for a cellulose-active AA9 LPMO carrying the HQY motif.^22^ On a related note, the presence of a tyrosine at position 3 and glutamate at position 2 does not exist naturally in AA10 LPMOs but is the most prevalent combination in (chitin-active) LPMOs in the AA11 family.

An approximately 5-fold increase in oxidase activity in the equivalent glutamine to glutamate mutant was also observed for a cellulose-active AA9 LPMO, *Nc*AA9C, carrying the HQY motif.^22^ The similarity of the mutational effects is remarkable considering that the catalytic domains of fungal *Nc*AA9C and bacterial *Ma*AA10B share only 24.8% sequence identity and considering that the extended copper environments between these two enzymes, i.e., beyond the HQY motif, vary. Combining the two studies, it is clear that the nature and the location of the headgroup of this residue are of crucial importance for copper reactivity, regardless of the type of LPMO. Hall et al., (2023)^22^ showed that the glutamine to glutamate mutation in *Nc*AA9C reduces the reduction potential and decreases the ratio between the reduction and reoxidation rates by 500-fold, providing an explanation for the increase in oxidase activity.

Above we have addressed the complexities of quantitatively assessing LPMO activity. The increased oxidase activity of the HEY mutant leads to higher LPMO activity under apparent monooxygenase conditions but also to increased enzyme inactivation. Increased enzyme inactivation has two possible causes: (1) H_2_O_2_ levels are too high, thus promoting the potentially damaging peroxidase reaction^66^ and/or (2) the mutation hampers the confinement of reactive intermediates that is needed to keep these intermediates from engaging in damaging off–pathway reaction within the enzyme–substrate complex. Indeed, work by Bissaro et al., (2020)^21^ on a chitin-active AA10 LPMO has shown the importance of a residue analogous to glutamine at position 2 in *Ma*AA10B for confining reactive oxygen species. Computational studies have pointed at a similar role for the glutamine in the HQY second sphere motif of *Ls*AA9A.^31^ Neutron diffraction studies^19,37^ have shown that second sphere residues may interact with emerging reactive oxygen species. The peroxygenase reactions described above show that the Q219E mutation affects the ability of *Ma*AA10B to use H_2_O_2_ productively and to avoid damage, especially in reactions with cellulose.

Unexpectedly, and of major interest, the effect of the Q219E mutation on LPMO performance was clearly substrate-dependent. Based on multiple experimental and computational studies,^34,38,67−69^ it has been pointed out that the bound substrate is a major determinant of the catalytic competence of an LPMO. To the best of our knowledge, the substrate-dependency of the effect of the Q219E mutation in *Ma*AA10B provides the first clear experimental example supporting this idea. It will be of interest to further explore the mechanistic basis of these substrate effects, for example through computational studies.

The strong and complicated impact of the substrate on LPMO catalysis is also apparent from the remarkable observation that both WT *Ma*AA10B and the HEY mutant have a higher affinity for PASC than for β-chitin, based on binding (Figure S4) or oxidase activity assays (Figure 4). The results depicted in Figure 4 show that PASC inhibits H_2_O_2_ formation to a much larger extent than β-chitin, for both enzyme variants, showing that PASC interacts more strongly with the catalytic domain, excluding it from the solvent and thus limiting the oxidase reaction. Nevertheless, the two variants clearly differ in terms of their preference for turning over chitin versus cellulose. These intriguing observations suggest that substrate affinities are affected by access to H_2_O_2_. In other words, while PASC may bind clearly better than β-chitin to the LPMO in the absence of H_2_O_2_, the situation may be different for the formation of productive ternary complexes with both substrate and H_2_O_2_.

All in all, this study shows that second sphere residues are important determinants of LPMO reactivity, both in isolation and combination with one another. Importantly, next to modulating the reactivity of the copper and the formation and fate of reactive oxygen species, these residues also affect substrate specificity. Thus, when exploring known and yet to be discovered LPMO functional diversity, both the extended substrate-binding surface and the configuration of the copper sites need to be considered.

## Supporting Information Available

Phylogenetic tree of AA10 LPMOs; primers used for site-directed mutagenesis of MaAA10B; theoretical extinction coefficients for all proteins purified in this study; prevalence of second sphere residue in cellulose-active AA10 LPMOs; structural superposition of second sphere residues in MaAA10B and ScAA10C; mutagenesis strategy; initial catalytic rates of MaAA10B variants; binding of full length and truncated MaAA10B to PASC and F062-chitin; and release of oxidized products by WT MaAA10B and the HEY and REY mutants in reactions supplemented with H_2_O_2_ and assessment of enzyme inactivation (PDF)

## Accession Codes

*Ma*AA10B, UniProt D9SZQ3*Sc*AA10C, UniProt Q9RJY2

## Notes

All data are contained within the manuscript.
