# Supplementary material for: Impact of the Copper Second Coordination Sphere on Catalytic Performance and Substrate Specificity of a Bacterial Lytic Polysaccharide Monooxygenase
Source: ACS Omega. 2024 May 15;9(21):23040–52. doi: 10.1021/acsomega.4c02666 (PMC11137697; doi:10.1021/acsomega.4c02666)
Supplement: Supplementary file 1 — ao4c02666_si_001.pdf [file ao4c02666_si_001.pdf]

Supporting Information for

Impact of the copper second coordination sphere on catalytic performance and substrate specificity of a bacterial LPMO

Kelsi R. Hall[1,2], Maja Mollatt[1], Zarah Forsberg[1], Ole Golten[1], Lorenz Schwaiger[3], Roland Ludwig[3], Iván Ayuso-Fernández[1], Vincent G.H Eijsink[1], Morten Sørlie[1*]

[1] Faculty of Chemistry, Biotechnology and Food Science, Norwegian University of Life Sciences (NMBU), 1432, Ås, Norway.

[2] Current address – School of Biological Sciences, University of Canterbury, Christchurch 8140, New Zealand.

[3] Department of Food Science and Technology, Institute of Food Technology, University of Natural Resources and Life Sciences, Vienna (BOKU), 1190 Vienna, Austria.

*Correspondence to: morten.sorlie@nmbu.no

This PDF file includes:

1. List of Supplementary Figures & Tables page 2
2. Supplementary Figures (1-5) and Tables (1-3) page 3-11
3. Supplementary References page 12

1. List of Supplementary Figures and Tables

Figure S1. Phylogenetic tree of AA10 LPMOs.

Table S1: Primers used for site directed mutagenesis of *Ma*AA10B.

Table S2: Theoretical extinction coefficients for all proteins purified in this study.

Figure S2. Prevalence of second sphere residue in cellulose-active AA10 LPMOs.

Figure S3. Structural superposition of second sphere residues in *Ma*AA10B and *Sc*AA10C.

Supplementary Discussion. Mutagenesis strategy.

Table S3. Initial catalytic rates of *Ma*AA10B variants.

Figure S4. Binding of full length and truncated *Ma*AA10B to PASC and β-chitin.

Figure S5. Release of oxidised products by WT *Ma*AA10B and the HEY and REY mutants in reactions supplemented with H_2O_2 and assessment of enzyme inactivation.

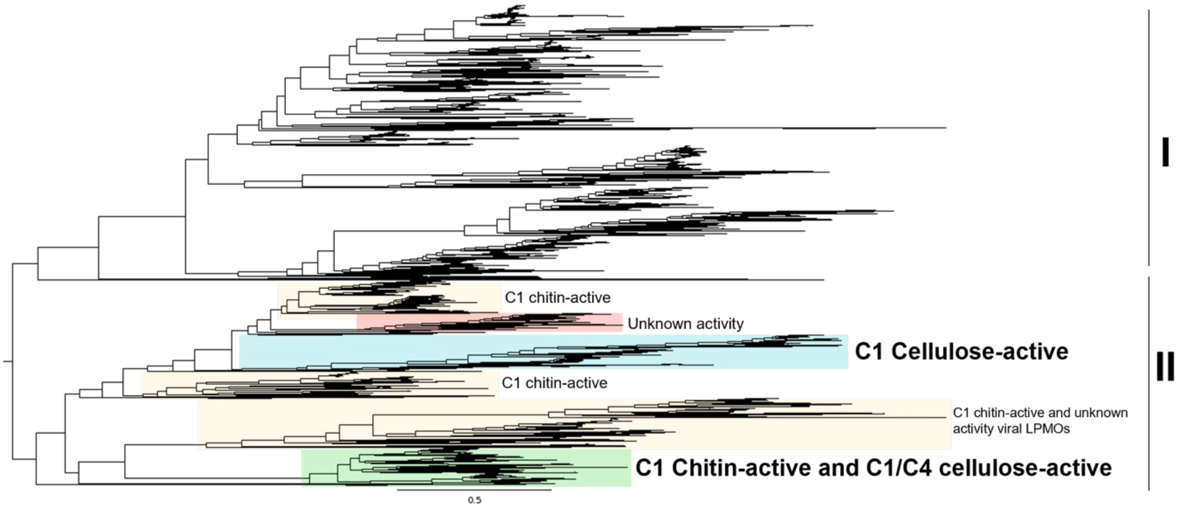

Figure S1. **Phylogenetic tree of AA10 LPMOs**. 3129 unique AA10 LPMO sequences were retrieved from dbCAN[1] (version 07262023) and aligned using MAFFT[2] under automatic selection of parameters. The tree was built using fasttree with default parameters. The overall topology of the tree consisted of two clades: Clade I, corresponding to C1 chitin-active LPMOs, and Clade II, with several activities. To identify functional subclades in clade II, sequences of known activity according to Forsberg *et al.,* (2019)[3] and Votvik *et al.,* (2023)[4] were added to the dataset. The LPMOs used in this study for assessing the occurrence of second sphere motifs appear in the blue and green subclades.

Table S1: Primers used for site directed mutagenesis of *Ma*AA10B. The underlined sequences show the mutated codon. Note that the template gene was codon-optimised for *E. coli* expression.

**Construct**	Forward primer (5'→3')	Reverse primer (5'→3')
*Ma*AA10B (HQ**F**)	CCAATCGTTTTACCTGTGCAGC<br>GATGTTGACTTTGGCGGTTCT	CACAGGTAAAACGATTGGTCCAG<br>GTGTGACGCCTGCCAGATGG
*Ma*AA10B (H**E**Y)	CACCTGGACGAATCGTATTACC<br>TGTGCAGCGATGTTGACTTTG	ATACGATTCGTCCAGGTGTGACG<br>CCTGCCAGATGGTATACACG
*Ma*AA10B (**R**QY)	GGCAGCGGTCAGGCCTGGACCA<br>ATCGTATTACCTGTGCAGCGA	CCAGGCCTGACCGCTGCCAGATG<br>GTATACACGACATGGCGGCC
*Ma*AA10B (H**EF**)	TGGACGAATCGTTTTACCTGTG<br>CAGCGATGTTGACTTTGGCGG	GGTAAAACGATTCGTCCAGGTGT<br>GACGCCTGCCAGATGGTATA
pRSETB backbone + CBM*	TGCAGCGATGTTGACTTTGGCG<br>GTTCTGGT	GCGACGATACGGCGTTGACATAT<br>GTATATCTC

* Variants which were ordered as gene fragments (RQF, REY, REF and REFex) were cloned into a pRSETB backbone which was amplified alongside the linker region and the CBM2.

Table S2: Theoretical extinction coefficients for all proteins purified in this study. Coefficients were calculated using the ExPASy ProtParam tool.

**Construct**	**Extinction Coefficient (M^{-1}. cm^{-1})**
WT *Ma*AA10B	93,765
*Ma*AA10B HQ**F**	92,275
*Ma*AA10B H**E**Y	93,765
*Ma*AA10B **R**QY	93,765
*Ma*AA10B H**EF**	92,275
*Ma*AA10B **R**Q**F**	92,275
*Ma*AA10B **RE**Y	93,765
*Ma*AA10B **REF**	92,275
*Ma*AA10B **REFex**	92,275
WT *Sc*AA10C	75,755

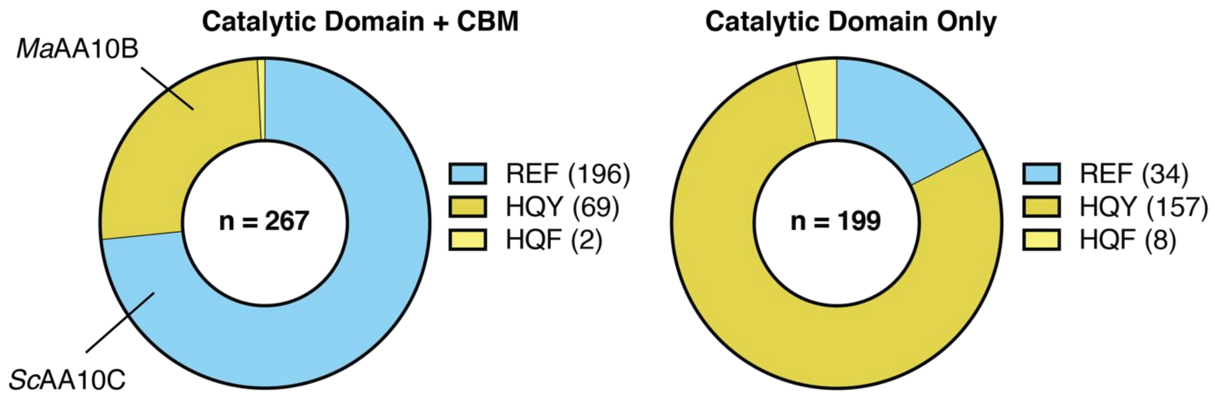

Figure S2. Prevalence of second sphere residue in cellulose-active AA10 LPMOs. All cellulose-active AA10 LPMOs (including those with mixed activity) from the CAZy database (n = 466) were aligned to determine the frequency of different amino acids at positions 1, 2 and 3 (see Figure 1). Prior to the alignment, the sequences were separated into two groups based on whether they contained a CBM or only a catalytic domain. The amino acid combinations found in *Ma*AA10B (HQY) and *Sc*AA10C (REF) are shown in darker yellow and blue respectively.

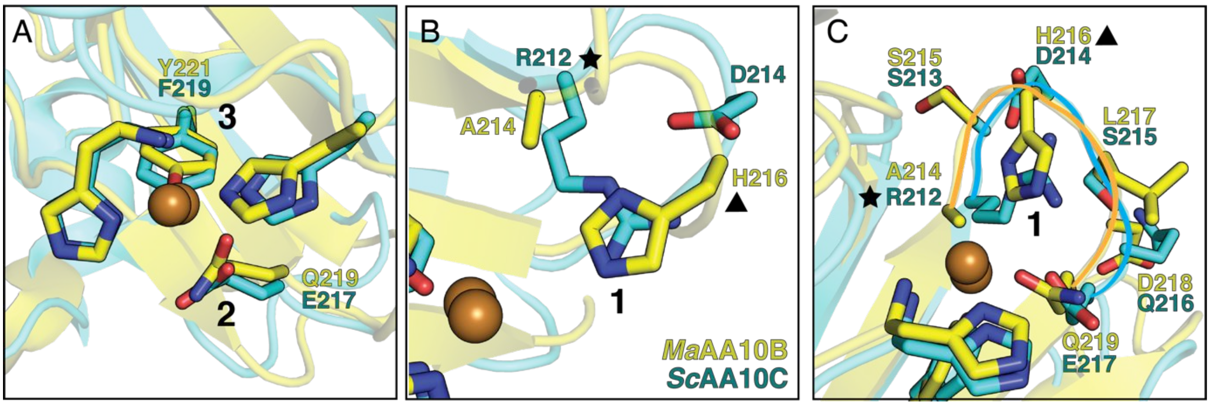

Figure S3. Structural superposition of second sphere residues in *Ma*AA10B (yellow carbons; PDB 5OPF) and *Sc*AA10C (blue carbons; PDB 4OY7). The structural alignment of *Ma*AA10B and *Sc*AA10C was performed using the align function in PyMOL. (A) View highlighting positions 2 (glutamine/glutamate) and 3 (tyrosine/phenylalanine). The histidine brace and bound copper ion of both enzymes are shown for reference. Side chains at position 1 are not shown for a clearer view of positions 2 and 3. (B) View highlighting position 1 (histidine/arginine). For clarity the histidine brace and the glutamate/glutamine at position 2 are not shown. The side chains of the histidine and arginine are in the same position in the active site, but the histidine (▲) originates from a different position in the main chain compared to the arginine (★). An aspartate (D214) occurs at the equivalent position of the histidine in *Sc*AA10C and an alanine (A214) is located at the equivalent position of the arginine in *Ma*AA10B. The closest distance between the headgroups of Arg^{212} and Asp^{214} in *Sc*AA10C is 2.8 Å. (C) View highlighting the loop regions in which the second sphere residues shown in panel B are located; the loops are shown as solid lines in yellow (*Ma*AA10B) or blue (*Sc*AA10C). In one of the *Ma*AA10B variants (REFex) the complete 214-219 loop was exchanged; see main text and the Supplementary Discussion below for more details.

Supplementary Discussion: mutagenesis strategy

Although the headgroups of the histidine (in HQY) and arginine (in REF) residues are in the same position in the active sites of *Ma*AA10B and *Sc*AA10C the main chains differ (Figure S3B). Consequently, the histidine could not be directly mutated to an arginine, as this would cause steric hindrance. Therefore, the histidine was mutated to a smaller amino acid, glycine, to make room for the side chain of an arginine, which was introduced to replace alanine at position 214. Ideally, the histidine residue in *Ma*AA10B would have been replaced with an aspartate, as this residue is present in *Sc*AA10C, but modelling studies indicated that this would lead to steric hindrance in arginine containing *Ma*AA10B, due to other differences in the loop regions of the two enzymes (Figures S3B and C). In addition to variants containing this double mutation (H216G/ A214R), referred to as RQY, REY, RQF and REF, one additional triple variant was generated, referred to as REFex. In this variant the complete loop from residues Ala^{214}- Gln^{219} (ASHLDQ), which includes His^{216} at position 1 and Gln^{219} at position 2, was replaced by the corresponding loop in *Sc*AA10C (residues Arg^{212}-Glu^{217}; RSDSQE), including Arg^{212} at position 1 and Glu^{217} in position 2. One motivation for this mutation was to investigate the importance of the salt bridge between Arg^{212} and Asp^{214}.

Table S3. Initial catalytic rates of *Ma*AA10B variants. The values provided are the initial rate of the release of oxidised products from PASC or β-chitin (μM/min) under apparent monooxygenase conditions (Figure 2A and B) and the initial rate of the oxidase activity (s^{-1}) (Figure 2C). The rates were determined by fitting the data by linear regression. These rates were used to calculate the fold change in activity reported in Figure 2D.

	**PASC (μM/min)**	**β-chitin (μM/min)**	**Oxidase (s^{-1})**
WT (HQY)	0.12 ± 0.004	0.32 ± 0.03	0.019 ± 0.0003
HQ**F**	0.019 ± 0.005	0.006 ± 0.002	0.0002 ± 0.00009
H**E**Y	0.65 ± 0.04	5.3 ± 0.5	0.1 ± 0.02
**R**QY	0.0032 ± 0.0006	0.01 ± 0.007	0.00013 ± 0.00008
H**EF**	0.017 ± 0.001	0.10 ± 0.006	0.0034 ± 0.0002
**RE**Y	0.18 ± 0.03	0.26 ± 0.03	0.033 ± 0.003
**R**Q**F**	0.0088 ± 0.0005	0.008 ± 0.004	0.0033 ± 0.0003
**REF**	0.016 ± 0.005	0.09 ± 0.01	0.0067 ± 0.0002
**REFex**	0.013 ± 0.004	0.012 ± 0.002	0.0084 ± 0.0004

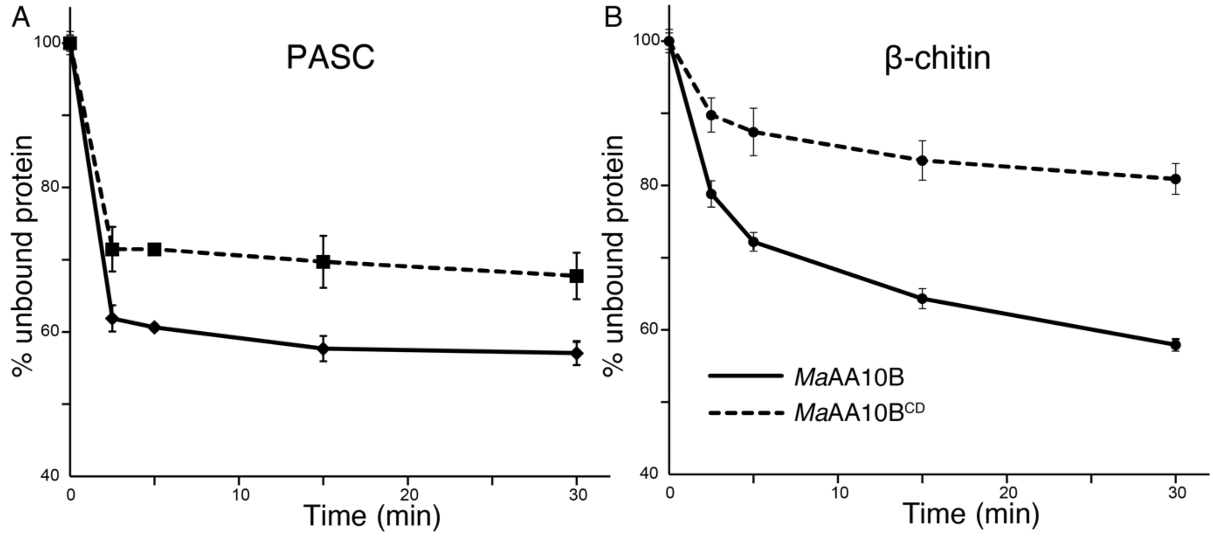

Figure S4. Binding of full length and truncated *Ma*AA10B to PASC (A) and β-chitin (B). Solid lines represent binding of full-length *Ma*AA10B (catalytic domain-linker-CBM2), whereas the dashed lines show binding for the catalytic domain of *Ma*AA10B (*Ma*AA10B^{CD}). 3 μM LPMO was incubated with 0.5 % (w/v) PASC (A) or 1% (w/v) β-chitin (B) in 20 mM sodium phosphate, pH 6.0, at 40 °C and 1000 rpm. Error bars show the standard deviation of triplicate reactions.

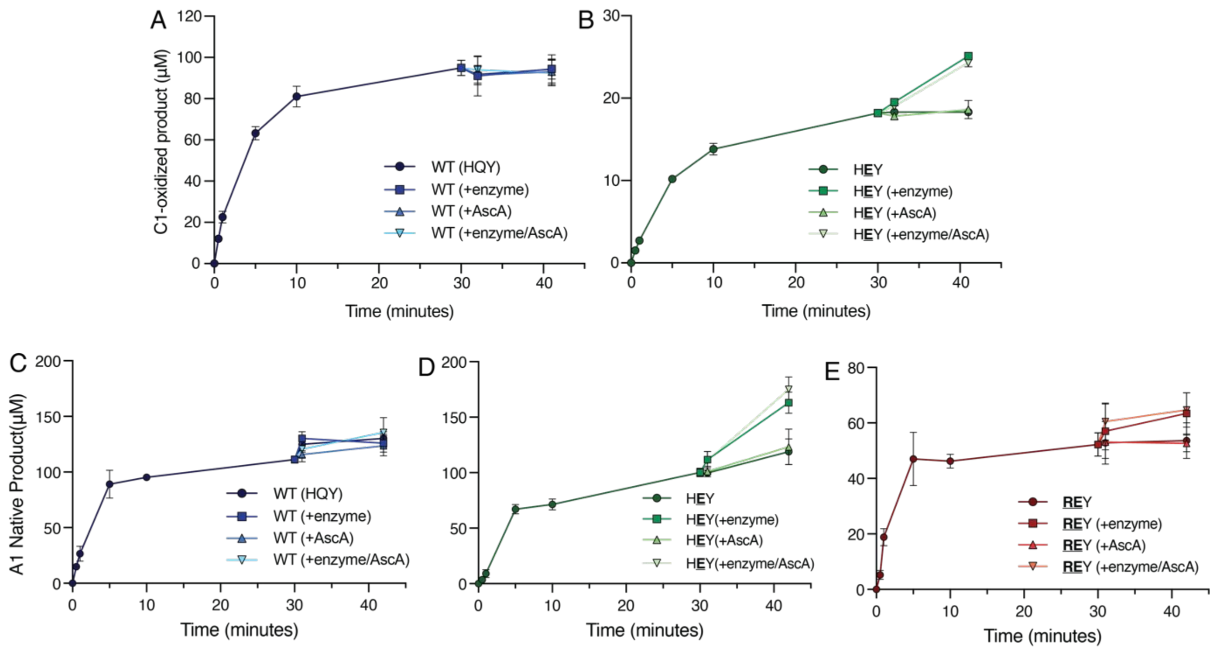

Figure S5. Release of oxidised products by WT *Ma*AA10B and the H<u>E</u>Y and R<u>EY</u> mutants in reactions supplemented with H_2O_2 and assessment of enzyme inactivation. All reactions were performed at 40 °C, 1000 rpm and contained 1 µM LPMO, 100 µM H_2O_2, and 0.1% (w/v) PASC (A, B) or 1% (w/v) β-chitin (C, D & E) in 20 mM sodium phosphate, pH 6.0. The reactions were initiated with the addition of 1 mM ascorbate. After 30 minutes, reactions were split and an additional 1 µM enzyme (squares, □), 1 mM ascorbate (triangles, ∆) or both enzyme and ascorbate (inverted triangle, ∇), or buffer only (circles, ○) were added to the reaction. Note that, within the time frame used here, addition of fresh enzyme to a reaction mixture that is depleted for H_2O_2 will not lead to detectable additional product formation. Error bars show the standard deviations of triplicate reactions.

Supplementary References.

(1) Zhang, H.; Yohe, T.; Huang, L.; Entwistle, S.; Wu, P.; Yang, Z.; Busk, P. K.; Xu, Y.; Yin, Y. dbCAN2: A meta server for automated carbohydrate-active enzyme annotation. *Nucleic Acids Res.* **2018**, *46*, W95-W101.

(2) Katoh, K.; Misawa, K.; Kuma, K.; Miyata, T. MAFFT: A novel method for rapid multiple sequence alignment based on fast Fourier transform. *Nucleic Acids Res.* **2002**, *30*, 3059-3066.

(3) Forsberg, Z.; Sørlie, M.; Petrovic, D.; Courtade, G.; Aachmann, F. L.; Vaaje-Kolstad, G.; Bissaro, B.; Røhr, Å. K.; Eijsink, V. G. H. Polysaccharide degradation by lytic polysaccharide monooxygenases. *Current Opinion in Structural Biology* **2019**, *59*, 54-64.

(4) Votvik, A. K.; Røhr, Å. K.; Bissaro, B.; Stepnov, A. A.; Sørlie, M.; Eijsink, V. G. H.; Forsberg, Z. Structural and functional characterization of the catalytic domain of a cell-wall anchored bacterial lytic polysaccharide monooxygenase from *Streptomyces coelicolor*. *Sci. Rep.* **2023**, *13*, 5345.
